# Supplementary material for: Getting More Out of Biomedical Documents with GATE's Full Lifecycle Open Source Text Analytics
Source: PLoS Comput Biol. 2013 Feb 7;9(2):e1002854. doi: 10.1371/journal.pcbi.1002854 (PMC3567135; doi:10.1371/journal.pcbi.1002854)
Supplement: Dataset S1 — GATE software. Dataset S1 bundled with this paper contains a distribution of GATE (or see http://gate.ac.uk/download/). (TGZ) [file pcbi.1002854.s001.tgz › plos-gate/doc/bugs.html]

GATE BUGS


## Reporting bugs

If you think you have found a bug in GATE, please:

- Make sure you are using the latest build of GATE. Many problems are found and
  fixed between major releases, and if you report a bug against a specific
  release of GATE the first thing we will ask you to do is try using the latest
  nightly build.
- Make sure you are using a supported version of Java. GATE 3.1 requires a Java
  runtime version 1.4.2 or later, GATE 4.0 beta 1 requires Java 5.0, and GATE 6.1 or later
  requires Java 6.0.
- Check the documentation, to make sure
  your environment is set up properly, and double check that you have followed
  the correct procedure (e.g. if using GATE as a library, that you have called
  Gate.init before using the Factory, that you have loaded the
  correct plugins, etc.).
- Check the mailing list archive and search the existing
  bug
  reports to see if your problem has already been reported (and possibly
  fixed).

Once you are sure you have found a bug, please let the appropriate person know
about it. Bugs in the core of GATE or in the plugins bundled with the GATE
distribution should be reported using the
SourceForge
bug tracker. Bugs in third-party plugins should generally be reported to
the plugin maintainer in the first instance, so the maintainer can
determine whether the bug is in their code or in the GATE core.
